# Enhancing the Adhesion Strength of Polymer-Based Joints via Atomic Layer Deposition Surface Modifications

**DOI:** 10.1021/acsami.5c04879

**Published:** 2025-05-29

**Authors:** Shachar Keren, Elina Yachnin, Noy Cohen, Tamar Segal-Peretz

**Affiliations:** † Department of Chemical Engineering, 26747 Technion−Israel Institute of Technology, Haifa 3200003, Israel; ‡ Department of Civil and Environmental Engineering, Technion−Israel Institute of Technology, Haifa 3200003, Israel; § Department of Materials Science and Engineering, Technion−Israel Institute of Technology, Haifa 3200003, Israel

**Keywords:** atomic layer deposition, adhesion, surface treatments, surface modification, mechanical properties, vapor phase infiltration

## Abstract

Enhancing the adhesion strength of polymer-based components is a critical challenge in advancing their use as structural and functional materials. Atomic layer deposition (ALD) offers a novel approach to address this challenge by enabling precise surface modifications that improve the adhesion performance. This work studies ALD-based surface modification of 3D-printed acrylonitrile butadiene styrene (ABS)-like single lap shear joints and investigates their adhesion performance. By depositing Al_2_O_3_, TiO_2_, and ZnO layers, we demonstrate significant improvements in shear strength, strain at failure, and toughness. Surface characterizations revealed that these enhancements stem from both chemical and physical modifications, including increased surface energy and the formation of wrinkled patterns that can facilitate mechanical interlocking. Different growth mechanisms led to the formation of distinct wrinkled and crease patterns; while Al_2_O_3_ and TiO_2_ grew on the polymer’s surface, ZnO grew within the ABS-like substrate via vapor phase infiltration (VPI). The surface morphologies and mechanical responses varied depending on the oxide type and number of ALD cycles. This work underscores the potential of ALD as a versatile surface treatment for improving adhesion performance in polymer-based materials and advancing bonding strategies for high-performance applications.

## Graphical abstract

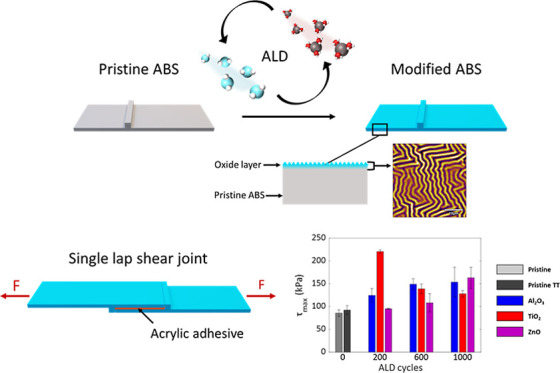

## Introduction

Polymer-based materials have become common structural materials in many applications, such as aerospace, automotive, and electronic devices due to their low cost, lightweight, and ability to tailor their properties and geometry to specific needs. Load-bearing structures and joints, traditionally made of metals and alloys, are increasingly being replaced, fully or partially, by polymer-based materials. While metal joints are usually joined together via welding, brazing, and bolting methods, joining polymer-based materials requires an alternative method. Adhesive joints enable bonding between polymer-based materials and multimaterial parts while attaining high mechanical performances and corrosion resistance. Moreover, adhesive bonding could be used as a cheap and fast technique for patch repairs, thus reducing the maintenance and time. Yet, using adhesives as a bonding technique could be challenging for some polymers due to their low surface energy, resulting in inferior adhesion.^1–3^ To address this issue, several commercial surface treatments are currently used to modify polymer and polymer-based composite surfaces, including mechanical treatments such as grinding or sandblasting and chemical treatments such as plasma, acid–base etching, and solvent cleaning.^4,5^ Over the years, numerous studies have developed surface modification techniques to enhance the adhesion of polymer-based materials, either by tuning surface roughness^6,7^ or through surface chemical modifications.^8,9^ Among these approaches, modifying the surface to create an outer oxide layer is particularly effective as it can improve the adhesive wettability and provide reactive hydroxyl groups that facilitate bonding with adhesive components.

A promising technique for surface modification and oxide deposition on polymers is through atomic layer deposition (ALD). ALD is a highly controlled vapor-phase deposition technique that enables growth of inorganic thin layers on top of various surfaces by exposing them to gaseous precursors in a cyclic process.^10,11^ A typical ALD process consists of a series of two alternating self-limiting half-reactions. First, a vapor precursor is introduced into a heated vacuum chamber at low pressure, where it reacts with the surface of a substrate to form a (sub)­monolayer. Once the surface is fully saturated, the chamber is purged to remove any unreacted precursor molecules and byproducts. The coreactant precursor is then introduced, reacting with the previously deposited monolayer to form a new surface ready for the next cycle. Repetition of this cycle results in the deposition of pinhole-free films, with atomic-level control over thickness and composition, even on high aspect ratio surfaces and complex surface geometries. ALD is a versatile technique, enabling the deposition of inorganic materials such as metals, metal oxides, nitrides, and sulfides on top of impermeable substrates.^12–14^

In addition, ALD can be harnessed to grow inorganic materials within permeable substrates such as polymers by tuning its conditions to drive the precursors into the polymer volume. This approach, also known as vapor phase infiltration (VPI) or sequential infiltration synthesis (SIS), involves extended exposure periods (typically tens of seconds to minuets), which enables the precursors to diffuse within the polymer substrate to grow inorganic material within polymers and form a hybrid material.^10,15,16^ The extent and nature of growth depend on the process conditions (temperature, exposure time, precursor partial pressure) as well as on the precursor-polymer solubility, diffusion, and chemical interaction.^15–21^

A recent study by Chen et al.^8^ highlighted the efficacy of ALD in enhancing the adhesion of polymer-based materials through surface modification. Their research demonstrated that using ALD to deposit aluminum oxide films can alter the polymer’s surface chemistry and energy, thereby improving adhesion and interfacial toughness without affecting surface roughness or bulk properties. However, subsequent studies have shown that depositing a stiff layer like Al_2_O_3_ onto a relatively elastic polymer substrate can modify the surface morphology by inducing wrinkle patterns.^22,23^

Inspired by the potential to harness both mechanisms—higher surface energy and increased surface roughness—our research examines the utilization of ALD as a surface treatment to enhance the adhesion of polymer joints, with a model system of 3D-printed acrylonitrile butadiene styrene (ABS-like) single lap shear. First, we examine the effect of ALD surface modification on the macro-scale adhesion performances of single lap shear joints. Specifically, we focus on three mechanical properties: shear strength, shear strain at failure, and toughness. Next, we demonstrate how through precise control of the number of ALD cycles and the type of oxide layer, one can tailor the surface chemical composition and morphology of the ABS-like substrates to optimize adhesion properties. We explore the observed variations in surface morphology and the underlying mechanisms of these modifications. Finally, we discuss the formation of unique surface structures on top of the TiO_2_-modified surfaces and their effect on the mechanical properties.

## Results and Discussion

To study the effect of ALD on the adhesion performances of polymer joints, we fabricated a model system, as schematically illustrated in Figure 1. ABS-like substrates were 3D-printed using a Stratasys PolyJet 3D printer (further details on the polymer substrates are provided in the Materials and Methods section). Next, their surfaces were modified by growing either Al_2_O_3_, TiO_2_, or ZnO on top of the ABS-like substrates via cyclic exposure to one of the precursors (trimethyl aluminum (TMA), diethyl zinc (DEZ), or titanium tetrachloride (TiCl_4_)) and the coreactant, H_2_O, at 80 °C. We have set the deposition process to an ALD continuous flow process, in which an ALD cycle was defined as a sequence of: 0.015 s pulse of water/10 s wait/0.015 s pulse of precursor/10 s wait. This relatively short exposure time is intended to limit VPI growth within the depth of the polymer, by limiting the diffusion time into the polymer volume.^15,18^ To ensure sufficient nucleation of oxide growth on the polymer surface and to tune the thickness of the oxide layers, we subjected the substrates to either 200, 600, or 1000 ALD cycles (Table S1, Supporting Information). Following the ALD process, two similar modified substrates were bonded with a soft commercial acrylic adhesive layer (VHB 4910, 3M) to form a single lap shear joint. VHB 4910 is a well-known commercial adhesive that has been extensively characterized in the literature for its viscoelastic behavior and detachment mechanisms, making it a suitable model system for studying polymer joints.^24–26^ In addition, it is easy to handle, and being supplied as a tape of fixed thickness, it ensures a consistent adhesive layer thickness across all samples. This consistency helps to eliminate variations that could otherwise influence the mechanical results.

**Figure 1:**
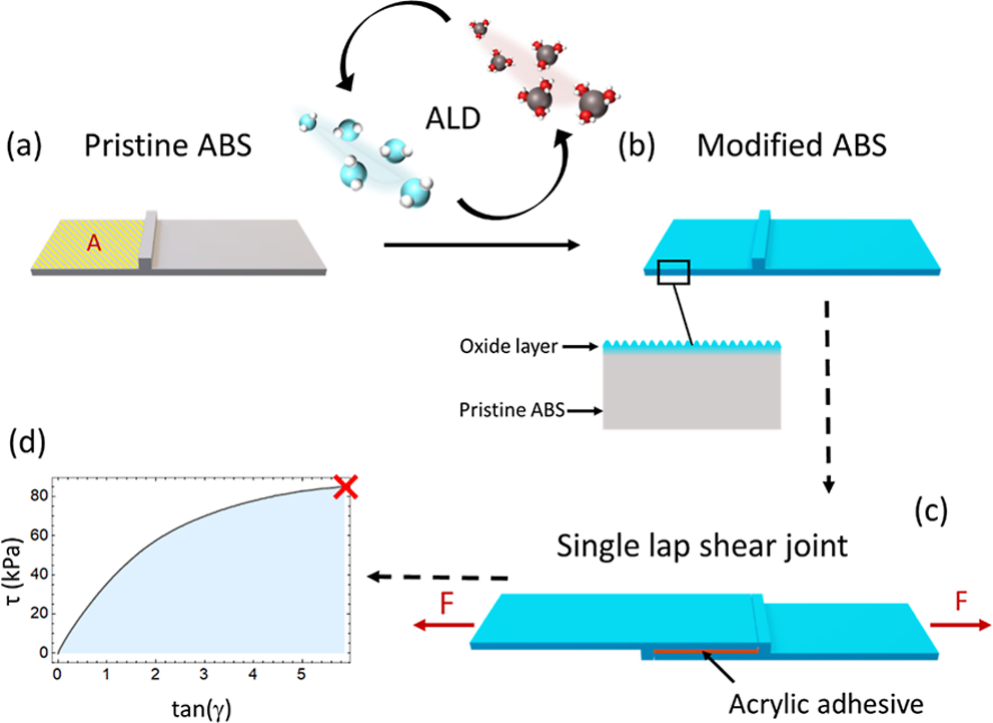
Schematic illustrations of the fabrication and mechanical characterization of an ABS-modified single lap shear joint with overlap adhesion area A (marked with yellow cross lines). (a,b) 3D-printed pristine ABS-like substrates were exposed to an ALD process to modify their surfaces by growing an Al_2_O_3_, ZnO, or TiO_2_ oxide layer. (c) Following the ALD process, two similar modified substrates were bonded using a soft commercial adhesive tape (VHB 4910, 3M) to assemble a single lap shear joint. The joint was subjected to uniaxial extension to probe the mechanical response using force-controlled mode. (d) Three mechanical characteristics were obtained: maximum shear strain (tan­(γ)_max_) and maximum shear strength (τ_max_) obtained at the failure point (marked as red X), and toughness (U_T_) obtained from integrating the area under the stress–strain curve.

To investigate the influence of the ALD process on the mechanical response of the adhesive, we designed a single lap shear joint, as shown in Figure 1. The adhesive is subjected to simple shear using force-controlled mode as described in our previous study and in the experimental section.^27^ Each type of lap shear was tested three times to ensure reproducibility, and the average of the repetitions was analyzed. The average shear-stress–strain curves and the numeric values of the mechanical properties can be found in the Supporting Information (Figure S1 and Table S2). Three mechanical quantities were obtained via shear measurements as described in Figure 1d: maximum shear strain (shear strain at failure) tan­(γ)_max_, maximum shear strength (shear stress at failure) τ_max_, and toughness *U*_*T*_. These characteristics are defined as

1 $$\tan\left(\gamma \right)=\frac{u}{t}$$

2 $$\tau =\frac{F}{A}$$

where *u* is the measured displacement, *t* = 1 mm is the thickness of the adhesive, *F* is the applied load, and *A* = 25.6 × 25.6 mm^2^ is the overlap adhesion area (Figure 1). The toughness of a material, *U*_T_, defined as the area under the stress–strain curve, characterizes the joint capacity to absorb energy prior to failure.

The mechanical characteristics obtained from the shear measurements are shown in Figure 2. First, we probed the effect of thermal postcuring on the mechanical properties by exposing pristine lap shears to a similar thermal profile as the 1000-ALD-cycles modified lap shears (∼12 h at 80 °C and ∼0.3 Torr). For convenience, we defined this type of lap shear as “Pristine TT” (indicating thermal treatment). The thermal treatment by itself led to a slight increase in the maximum shear strain and toughness of the pristine TT compared with the pristine samples. This could be due to an increased cross-link density and the reduced fraction of relatively short chains on the surface as a result of a thermal postcuring.^28–30^

**Figure 2:**
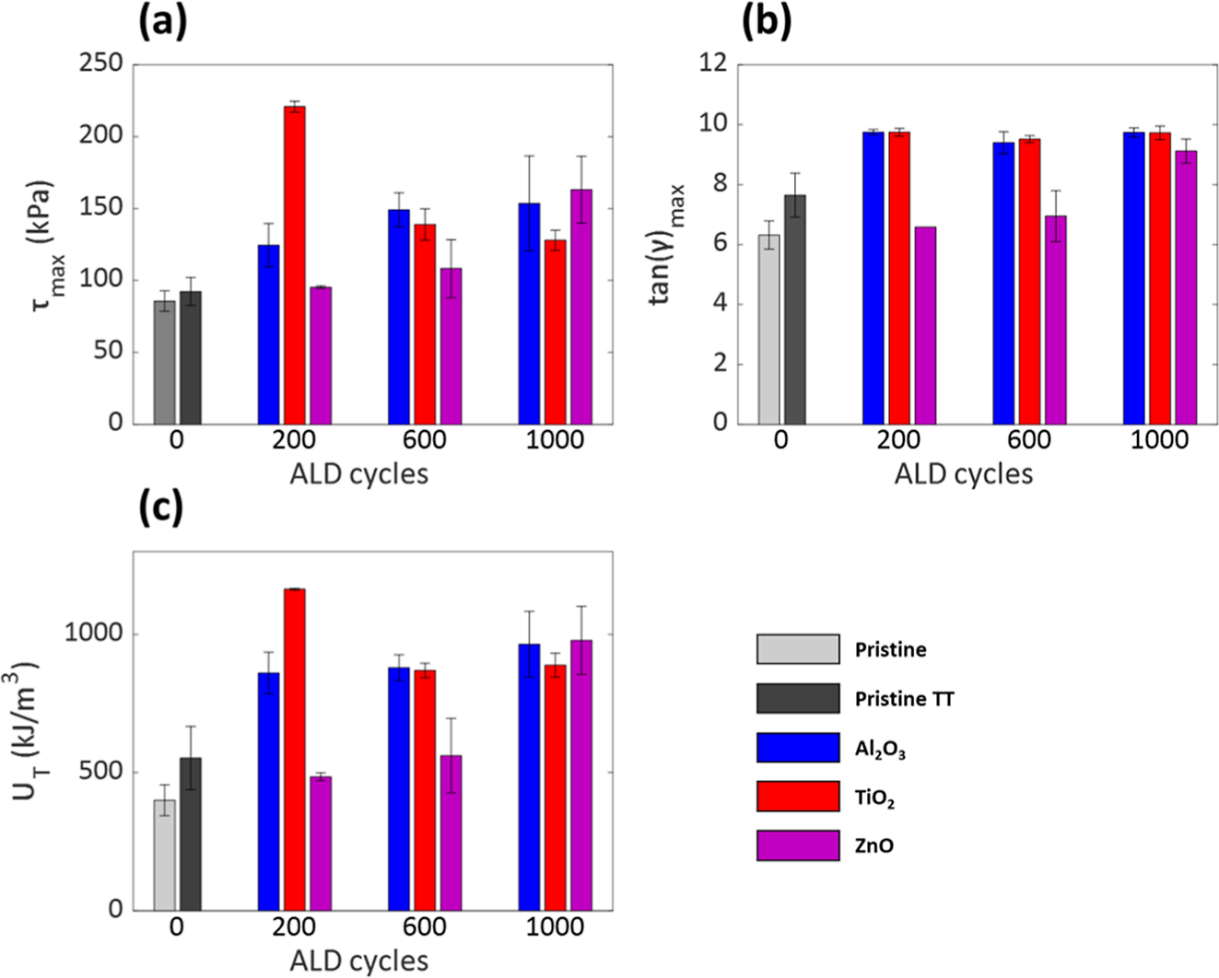
The mechanical properties of pristine, pristine after thermal treatment (TT), and ALD-modified lap shears, obtained via shear measurements: (a) shear stress at failure τ_max_, (b) shear strain at failure Tan­(γ)­max, and (c) toughness U_T_.

Next, we probed the effect of ALD modification on the adhesion performances compared to the Pristine TT. The growth of all oxide layers, Al_2_O_3_, ZnO, and TiO_2_ via ALD on top of the ABS-like surfaces has proven to enhance the mechanical properties of the adhesive joints, as shown in Figure 2. The Al_2_O_3_-modified lap shears show enhanced adhesion performances in all three mechanical properties after 200 ALD cycles. We found that increasing the number of ALD cycles to 600 and 1000 does not significantly improve adhesion performances, as the differences in the average mechanical properties fall within the range of standard deviations. For the TiO_2_-modified lap shears, a significant adhesive improvement is achieved after 200 ALD cycles with a significant increase in shear strength and toughness. It is also worth noting that this modification presents the highest mechanical properties of all the modified lap shears, with an enhancement of ∼140%, ∼27% and ∼110% in τ_max_, tan­(γ)_max_, and *U*_*T*_, respectively, compared to pristine TT. Interestingly, additional ALD cycles resulted in decreased strength and toughness, compared to the 200 cycles modification, but improved adhesion performances, compared to the pristine TT. We discuss this phenomenon in the last section of this study. No significant mechanical enhancements were observed after 200 and 600 ALD cycles for the ZnO-modified lap shears. Significant improvements in all three mechanical properties were observed only after 1000 ZnO cycles.

These results demonstrate a significant improvement in the macro-scale adhesion properties of ALD-treated ABS-like joints compared to nontreated ones. Interestingly, the variations in mechanical properties suggest that the oxide layer type and the number of ALD cycles play crucial roles in enhancing adhesion performance. In the following sections, we characterize the surfaces of the pristine and modified substrates to understand the underlying mechanisms of these variations. We specifically focus on the surface morphology and the surface energy correlated to the chemical composition.

We employed scanning electron microscopy (SEM) combined with energy-dispersive X-ray spectroscopy (EDS) to validate the growth of the oxide layers on the ABS-like substrate surfaces. The SEM-EDS images (Figure 3) not only confirm the successful growth of the oxide layers but also reveal a wrinkled surface morphology. While the pristine and Pristine TT had a smooth surface morphology (Figures S2), all ALD-treated ABS-like surfaces displayed wrinkled morphologies with varying patterns, indicating that the ALD treatment on ABS-like substrates induces distinct physical modifications at the microstructural level. The mechanism and properties of wrinkle formation will be discussed in detail in the final section of the paper. These wrinkled morphologies increase the surface roughness, potentially enhancing the available surface area for adhesion. Consequently, ALD treatment may induce both chemical and physical changes that contribute to the lab shear mechanical properties.

**Figure 3:**
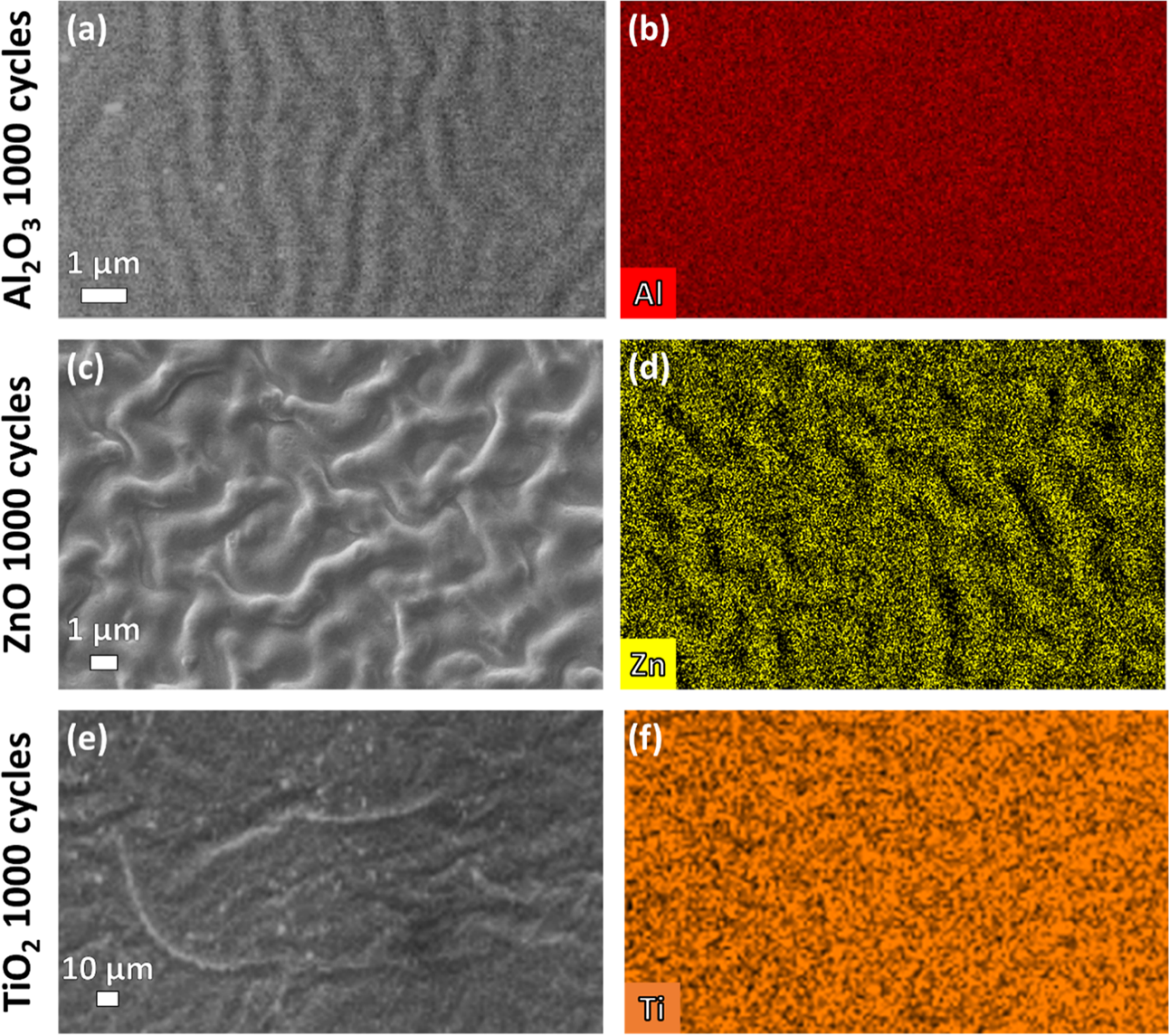
Top-down SEM images (left) combined with EDS analysis (right) of an Al_2_O_3_-modified ABS-like substrate (a,b), ZnO-modified ABS-like substrate (c,d), and TiO_2_-modified ABS-like substrate (e,f) modified with 1000 ALD cycles.

To estimate the surface energy of the substrates, we measured the contact angles using two liquid probes. We calculated the total surface energy using the Owens and Wendt model (see Method section).^31,32^ As shown in Figure 4 and Table S3, the results indicate that ALD surface treatment slightly increases the surface energies, mainly due to an increase in the polar component of the surface energies. While these results suggest a possible modification in the surface chemical composition due to the growth of oxide layers on the ABS-like substrate surface, they do not solely explain the mechanical enhancements of the ALD-modified ABS-like. Moreover, the surface energies do not reflect the variations in the mechanical behavior that seem to be affected by the number of ALD cycles and the type of grown oxide. Thus, we turn to examine the surface morphology.

**Figure 4:**
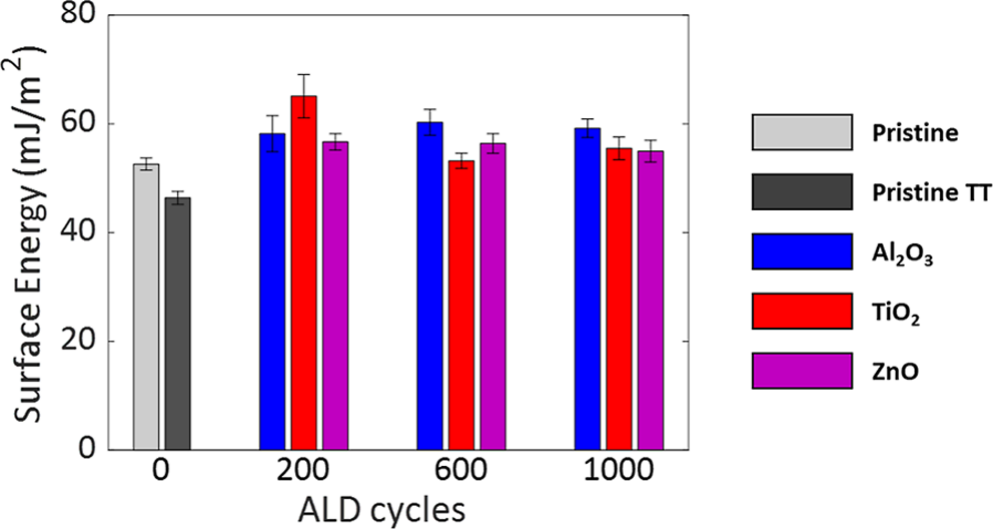
The surface energies of the pristine and modified substrates calculated using the contact angle measurements and Owens and Wendt model.

To further investigate these surface morphologies, we employed atomic force microscopy (AFM). In agreement with our SEM observations, the nonmodified (pristine and pristine TT) ABS-like substrates exhibited relatively smooth surfaces with no distinctive morphology, while all the ALD-treated ABS-like surfaces had maze-shaped wrinkling patterns (Figures 5a–h and S3). However, notable differences were observed between the ALD-modified surfaces. The patterns on the Al_2_O_3_- and TiO_2_-modified surfaces consist of elongated and continuous lines, whereas ZnO has a denser pattern with fragmented lines. These morphological variations likely contribute to the differences in adhesion and mechanical properties observed among the samples. To quantify these morphological differences and gain deeper insights into their potential impact on adhesion performance, we analyzed two key parameters of the wrinkling patterns: the average wavelength (λ) and the average amplitude (Α) (Figure 5i,j). The wavelength (λ) represents the distance between two consecutive peaks or valleys and provides information about the pattern density. The amplitude (Α), which indicates the height of the surface features, offers insight into the depth of the mechanical interlocking potential. By measuring these characteristics, we aimed to establish correlations between the specific surface morphologies induced by different ALD treatments and the resulting adhesion properties. The numerical values of the average wavelengths and amplitudes can be found in the Supporting Information (Table S4).

**Figure 5:**
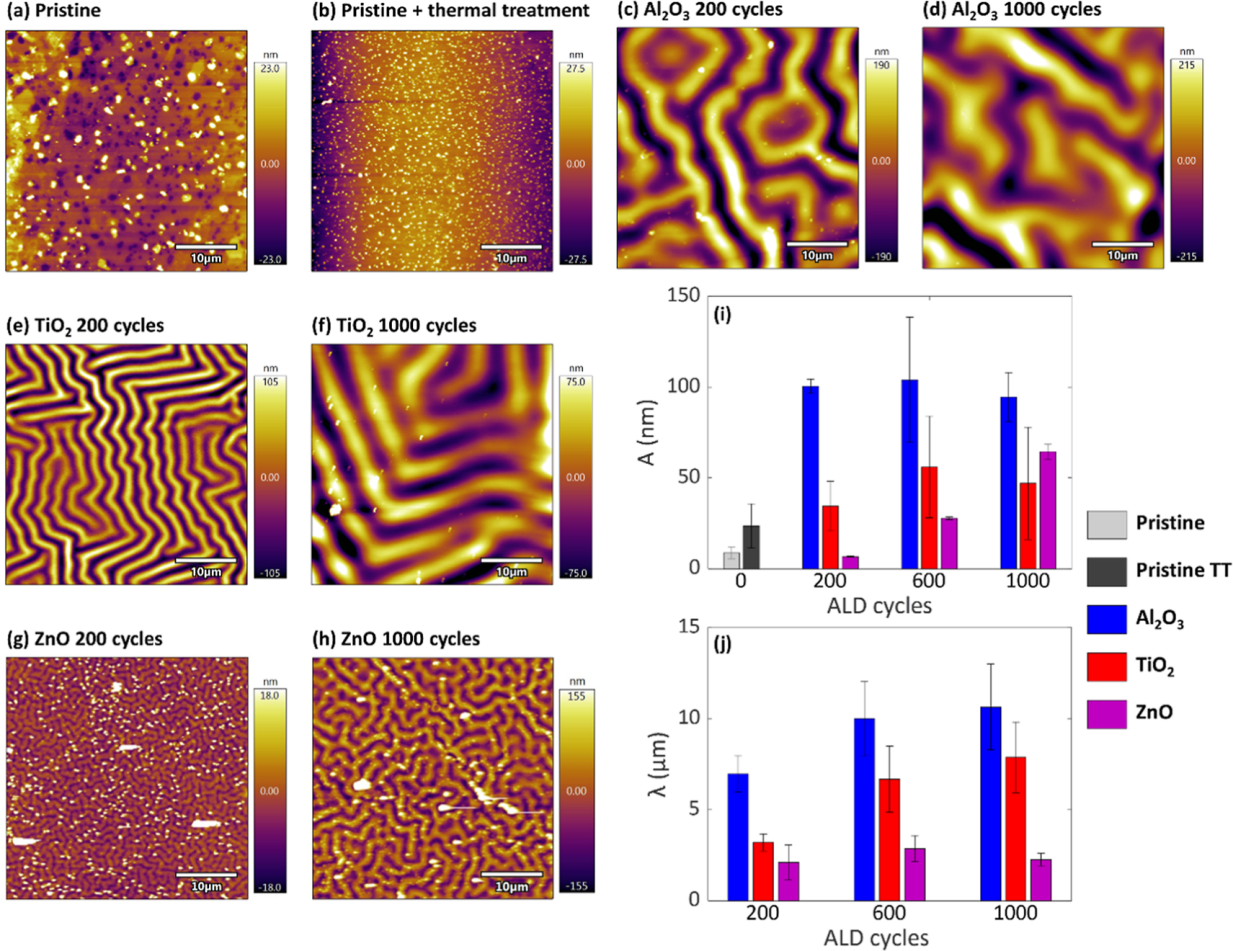
(a–h) AFM scans of the ABS-like substrates at their pristine stages and after surface modifications with an oxide layer: (a) pristine–no surface modification, (b) pristine (thermal treatment)–no surface modification but similar thermal exposure profile as the 1000 ALD samples, (c) Al_2_O_3_ layer grown with 200 ALD cycles, (d) Al_2_O_3_ layer grown with 1000 ALD cycles, (e) TiO_2_ layer grown with 200 ALD cycles, (f) TiO_2_ layer grown with 1000 ALD cycles, (g) ZnO layer grown with 200 ALD cycles, (h) ZnO layer grown with 1000 ALD cycles. (i,j) The average surface characteristics of the wrinkle patterns obtained from the AFM scans: (i) wavelength (λ) and amplitudes (Α).

The amplitudes showed different trends among the modified surfaces. At the pristine stage, the surfaces present roughness amplitudes of 8.7 ± 3.2 nm and 23.5 ± 12.2 nm (pristine and pristine TT, respectively). For Al_2_O_3_-modified surfaces, the amplitude of the formed pattern increases to 100.5 ± 3.9 nm after 200 ALD cycles, and it remains close to constant regardless of the number of ALD cycles. For the TiO_2_-modified surfaces, the amplitude slightly increases to 34.4 ± 13.7 nm after 200 ALD cycles. With further increases in the number of ALD cycles to 600 and 1000, the average amplitude rose to 56.0 ± 28.0 and 47.0 ± 30.9 nm, respectively. However, the large ranges of standard deviations at a large number of ALD cycles indicate considerable variability, suggesting that the amplitude might not increase significantly between 200 and 1000 cycles. This point is further discussed in the following section. In contrast, the amplitude of the ZnO-modified surfaces after 200 cycles shows a similar value as the smooth pristine (6.6 ± 0.3 nm) but modestly increased with additional ALD cycles- 27.7 ± 0.8 and 64.4 ± 4.1 nm after 600 and 1000 ALD cycles, respectively.

Regarding the wavelength, since the pristine and pristine TT did not present any wrinkling morphologies, we discuss the wavelength of the modified substrates. For Al_2_O_3_-modified surfaces, the wavelength after 200 ALD cycles was 6.96 ± 1.00 μm. Increasing the number of cycles above 200 slightly increased the wavelength to 10.00 ± 2.04 μm and 10.64 ± 2.36 μm after 600 and 1000 ALD cycles, respectively. For the TiO_2_-modified surfaces, the wavelength after 200 ALD cycles was 3.20 ± 0.47 μm. Increasing the number of cycles above 200 increased the wavelength to 6.67 ± 1.81 μm after 600 ALD cycles and 7.87 ± 1.94 μm after 1000 ALD cycles. Interestingly, the ZnO-modified surfaces present a smaller λ than the other two oxides, which remains constant regardless of the number of ALD cycles (∼2.1–2.9 μm).

Following the characterization of the surface morphologies, several critical questions arise from these observations. First, what mechanisms drive the formation of these morphologies? Second, why do ZnO-modified surfaces exhibit distinct morphologies compared to Al_2_O_3_- and TiO_2_-modified surfaces?

To answer these questions, we examined the surface wrinkling of the substrate. Previous works reported the formation of such patterns in bilayered systems with a mismatch between the properties.^6,7,22,23,33–42^ Specifically, when a bilayered structure is subjected to an external stimulus such as osmotic pressure, swelling, heating, mechanical stretching, or compression, it experiences mechanical stresses. These stresses, combined with the mismatch in the mechanical properties between the layers, lead to surface buckling instabilities. For example, Chen et al.^22^ have studied the formation of spontaneous random wrinkles on poly­(dimethylsiloxane) (PDMS) surfaces due to Al_2_O_3_ growth via ALD. This study showed that random wrinkles spontaneously form at the end of the ALD process, when the PDMS substrates are cooled back to room temperature. The mismatch of thermal expansion coefficients between the polymer substrate and the Al_2_O_3_-rich layer causes strain mismatches, forming compressed stresses released by surface wrinkling. Similar phenomena were also observed in other non-ALD systems.^6,7,23,33–41^ Similarly, in our oxide-coated ABS-like substrates, the cooling stage after the ALD process and the mismatch between the thermal expansion coefficients of the ABS-like substrate and the oxide layers lead to the formation of wrinkled patterns. Interestingly, the morphological variations observed in our modified surfaces, namely, the high wrinkle wavelength when Al_2_O_3_ and TiO_2_ were deposited versus lower wavelength with ZnO, could be related to the oxide growth profile, resulting in different surface wrinkling instabilities. Previous studies have suggested that systems that undergo swelling are more likely to present crease pattern that would appear as discontinuous and sharp fold pattern like in the ZnO-modified substrates,^33,35,41,43^ while systems with modulus mismatch between the layers are most likely to present wrinkling patterns that appear continuous, smooth and wavy, like in the Al_2_O_3_ and TiO_2_-modified substrates.^35,36,41^

To further investigate the effect of composition on the formation of wrinkles, we cross-sectioned the 1000 cycles-modified ABS-like surfaces (Al_2_O_3_, TiO_2_, and ZnO) using plasma-focused ion beam milling (PFIB) SEM. We then directly probed the oxides’ spatial distribution, morphology, and thickness using high-angle annular dark field scanning TEM (HAADF–STEM) combined with energy-dispersive X-ray spectroscopy (EDS) elemental analysis (Figure 6).

**Figure 6:**
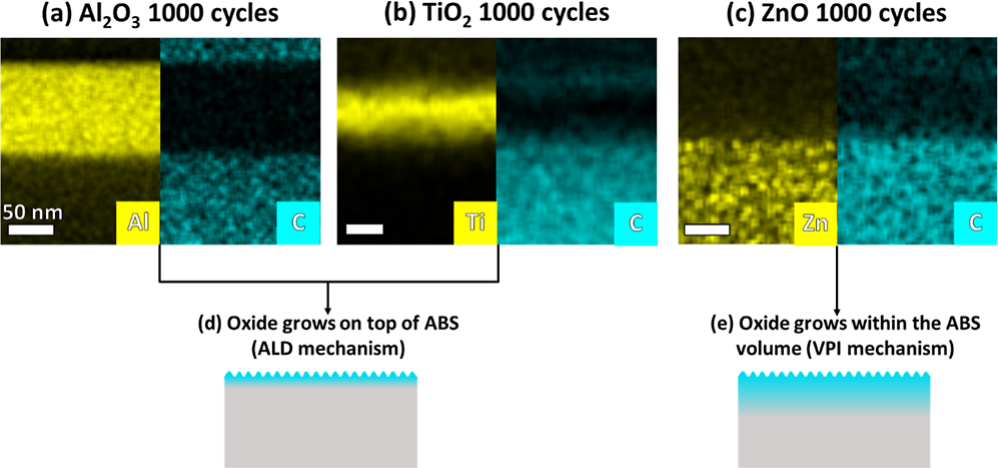
Cross-sectional STEM-EDS elemental maps of ABS-like substrates after 1000 ALD cycles with (a) Al_2_O_3_, (b) TiO_2_, and (c) ZnO modification layers on top. The comparison between the Al, Ti, and Zn signal and the C signal shows that the Al_2_O_3_ and TiO_2_ layers grew mainly on top of the ABS-like surface, with negligible infiltration into the ABS-like volume while ZnO grew mainly inside the ABS layer, as schematically illustrated in d,e.

The cross-sectional images in Figure 6 show different growth trends between the oxides. The comparison between the carbon signal, which stems from the ABS-like substrate, and the metal signals, which stem from the oxides, indicates that Al_2_O_3_ and TiO_2_ grow mainly on the ABS-like surface, with thicknesses of ∼110 and ∼120 nm, respectively, with negligible overlap between the oxide layer and the ABS-like substrate. The ZnO growth, on the other hand, completely overlaps with the ABS-like substrate, indicating that a hybrid layer with a thickness of ∼2.5 μm has formed (Figure S4, Supporting Information). These results imply that the ABS-like substrate reacts differently with the various precursors. Since VPI kinetics is governed by the diffusion of the precursor into the polymer and its reaction with it,^15,17^ a specific precursor-polymer pair will typically result in diffusion-limited growth or reaction-limited growth.^44,45^ Diffusion-limited growth occurs when the reactant diffusion into the polymer is slower than the chemical reaction rate. In short exposure times, this will lead to oxide growth near the polymer’s surface. In contrast, reaction-limited growth occurs when the chemical reaction rate is slower than the diffusion of the precursors. In short exposure times, reaction-limited growth will yield deeper diffusion into the substrate and, hence, growth within the polymer volume.

The photopolymers used for 3D printing contain acrylic monomers and oligomers, known to contain ester groups (see Materials and Methods section). Both TMA and TiCl_4_ are known to coordinate with ester groups.^46,47^ Thus, it is likely that the combination of relatively short ALD exposure periods combined with a fast chemical reaction between the precursor and the polymer results in a predominant diffusion-limited growth that mainly occurs at the substrate surface. As the number of cycles increases, the oxide layer hinders the penetration of precursor molecules into the polymer, leading to predominant growth of outer oxide layer, similar to a classic ALD process.^15,48^ On the other hand, DEZ has low Lewis acidity, which reduces its binding tendency toward the Lewis basic ester moieties, reducing ZnO nucleation on the ABS-like surface during early cycles.^19,49^ The low reactivity results in reaction-limited growth kinetics that facilitates deeper infiltration of DEZ into the polymer volume, enabling the growth of a hybrid ABS-ZnO layer in a mechanism similar to that in VPI (see Figure S5, Supporting Information).

In the case of Al_2_O_3_ and TiO_2_ deposited on ABS-like substrates, the formation of a rigid oxide layer on the compliant ABS-like substrate induces a modulus mismatch, resulting in the development of an elongated wrinkled pattern.^35,36,41^ While an increase in wavelength was observed, no significant change in amplitude occurred, likely due to surface inhomogeneity introduced by the 3D printing process. For both materials, the formation of wrinkles in ALD-modified ABS-like contributed to enhanced mechanical properties. For Al_2_O_3_, this improvement remained stable with increasing ALD cycles and greater oxide thickness. In contrast, TiO_2_ exhibited the highest mechanical performance at 200 ALD cycles, with a decrease at higher cycle numbers. We attribute the high performance of the 200 cycles TiO_2_ coating to an optimal combination of surface energy, roughness, and mechanical interlocking at the adhesive interface. However, the intrinsic properties of the oxide layers such as the chemical composition, crystalline structure, and interfacial bonding with the substrate can also have a significant role in the mechanical response of the adhesive joints.^50,51^ The decline in performance with additional TiO_2_ cycles is attributed to the unexpected growth of orchid-like TiO_2_ structures on the ABS-like surface (Figures S5 and S6), which due to their hollow structure and poor adhesion to the ABS-like surface may create weak boundary layer with the adhesive and negatively impact the mechanical performance. We note that the formation mechanism of these structures is unclear and beyond this work’s scope. However, we suspect it might be related to the formation of HCl reaction product, and the accumulation of chlorinated organic byproducts during the TiO_2_ ALD process, particularly at higher cycle numbers (see Figure S6, Supporting Information).^47,52^ In addition, since the deposition was conducted at 80 °C, below the established ALD temperature window for the TiCl_4_/H_2_O process, it is plausible that CVD-like reactions, including TiCl_4_ condensation, occur under these conditions. This could lead to the nucleation of nonuniform, particle-like features such as the orchid-like structures observed after 600 and 1000 cycles. Supporting this hypothesis, we note that the measured growth per cycle (GPC) at 80 °C is 0.070–0.084 nm, which is significantly higher than the ALD GPC of ∼0.048 nm typically observed at temperatures above 150 °C (Table S1, Supporting Information). These structures were clearly visible in SEM imaging (Figures S5 and S6).^52^ Furthermore, SEM-EDS (Figure S6) indicates high chlorine content in these regions, suggesting that the material may not be stoichiometric TiO_2_ but rather contains residual precursor species or intermediate phases formed under nonideal ALD reaction conditions.

For the ZnO-modified surfaces, the growth of ZnO within the polymer is likely to induce swelling of the polymer, leading to the formation of crease pattern morphology. An increase in the average amplitude was observed with increasing ALD cycles, while the wavelength remained constant. This behavior together with the formation of creases pattern indicate that the increase of the amplitude is likely driven by an increase in strain due to swelling,^48,53^ rather than a direct enhancement of the elastic modulus, as previously reported. The moderate yet consistent increase in amplitude with ALD cycles correlates to enhancement of the mechanical properties, suggesting that the crease pattern roughness enhances the adhesion through mechanical interlocking.

## Conclusions

In this work, we demonstrate the potential of ALD as an effective surface modification technique to enhance the adhesion performance of polymer-based materials. Through precise control over ALD cycles and the selection of metal oxides, we have shown significant improvements in shear strength, strain at failure, and toughness. Surface characterizations revealed that these enhancements are attributed to both chemical and physical modifications, including increased surface energy and the formation of wrinkled patterns that promote mechanical interlocking. Notably, the mechanical responses of the modified joints varied, depending on the type of oxide and the number of ALD cycles, highlighting the importance of optimizing these parameters. Thin coatings of TiO_2_ have shown the highest performance and can potentially be scaled up to meet the needs of specific applications. This work demonstrates the potential of ALD to enhance adhesion in polymer-based materials for high-performance applications. Future studies on the ALD-surface modification could facilitate the development of more durable and versatile bonding strategies.

## Materials and Methods

### 3D Printing

All of the single lap shear joint substrates were 3D-printed from a commercial ABS-like material (RGD515 and RGD531) with a glassy finish by using OBJET260 CONNEX 3 (Stratasys). The exact chemical composition of the polymer is unknown, and characterizing the raw materials or final cross-linked polymer was significantly challenging. However, the technical data sheet of the materials provided by the printer manufacturer indicates that the photopolymers used for 3D printing contain acrylic monomers and oligomers.

The substrates were designed with a thickness of 2 mm, a total length of 70 mm, a width of 25.6 mm, and an overlap adhesion area of 25.6 × 25.6 mm^2^. All substrates were washed from the supporting layer used in the printing process, were dried with a “clean room” cloth, and kept in a clean and dry environment (N_2_ atmosphere).

### Atomic Layer Deposition

All ALD processes were performed in a commercial ALD system (Savannah S100, Veeco) at 80 °C using one of the precursors (TMA/DEZ/TiCl_4_) and water as coreactant. For each ALD process, six substrates (three single lap shear joints) were placed in the ALD. The process included a 6 h stabilization step under 20 sccm of N_2_ flow at 0.3 Torr prior to the process. An ALD cycle was defined as a sequence of: 0.015 s pulse of water/10 s wait/0.015 s pulse of precursor/10 s wait. After a precursor pulse, the initial pressures were measured to be ∼ 0.8 Torr. During the process, the chamber valves were open with a constant flow of 20 sccm of N_2_. The samples were sequentially exposed to 200, 600, or 1000 ALD cycles. During the ALD processes, Silicon wafers (<100>, resistivity 0.005 Ω·cm) were used to monitor the thickness of the oxide layers.

### Assembly of a Single Lap Shear Joint

As described in our previous study, two similar ABS-like substrates were bonded together with 1 mm thick soft adhesive (VHB 4910, 3M).^27^ Note that the bonding occurred immediately after the ALD process, to avoid surface contamination. Also, special care and precautions were taken to ensure alignment and prevent air bubble entrapment or cavities in the adhesive interface. Specifically, after the adhesive was attached to the bottom substrate, a clean spatula was used to tighten the adhesive layer by pressing the release liner against a hard surface. Once the release liner was peeled from the clear adhesive, the specimen was examined for any visible bubbles, defects, or contamination. Next, a second ABS-like substrate was gradually attached to prevent bubble entrapment. To ensure good contact, manual pressure was applied to the assembled lap shears, followed by a tightening using four grippers for a minimum dwell time of 48 h.

### Uniaxial Extension Measurements

To examine the mechanical response of the adhesive layer under shear, a single lap shear joint was subjected to a uniaxial extension at room temperature by using an Instron 5943. Mechanical tests were performed in force-controlled mode with a rate of 0.1 N/s to simulate quasi-static loading, as described in our previous study.^27^ It is emphasized that due to the stiffness of the ABS-like materials, the deformations of the substrates are assumed to be negligible, such that the only deformation is due to the soft adhesive. Each experiment was performed three times to ensure that any contamination, if it exists, is negligible and the results are reproducible. The averages are presented.

### Contact Angle Measurements

ABS-like samples with a thickness of 2 mm, length of 50 mm, and width of 50 mm were 3D printed and modified via ALD, similar to the lap shear substrates. A goniometer (DSA25E, KRUSS) measured static contact angle using deionized water and diiodomethane as polar and dispersed liquid probes. For each measurement, a 10 μL drop of the liquid probe was dripped on the substrate surface, and the average of three measurements was calculated. Special care and precautions were taken to avoid contamination and ensure the symmetry of the drop. Assuming that the total surface energy can be divided into components representing different surface interactions (polar and dispersive), the surface energy components were calculated using relationships derived from the Owens and Wendt model^31,32^

$$\gamma =\gamma_{S}^{D}+\gamma_{S}^{P}$$

$$\left(1+\cos\theta \right)\gamma_{L}=2\left(\sqrt{\gamma_{S}^{D}\gamma_{L}^{D}}+\sqrt{\gamma_{S}^{P}\gamma_{L}^{P}}\right)$$

where γ is the total surface energy, θ is the contact angle, γ_L_ is the surface tension of the testing liquid (taken from the literature),^54^ γ_S_^D^ and γ_S_^p^ are the disperse and polar components of the sample surface energy (respectively), and γ_L_^D^ and γ_L_^P^ are the disperse and polar components of the testing liquid surface tension (taken from the literature).^54^ The average contact angles for the liquid probes, as well as the calculated dispersive and polar components of the surface energy, are shown in Table S2, Supporting Information.

### Atomic Force Microscopy

Before AFM measurements, ABS-like samples with a thickness of 2 mm, length of 10 mm, and width of 10 mm were 3D printed and modified via ALD, similar to the lap shear substrates. AFM measurements were conducted using an “Asylum Research/Oxford Instruments MFP-3D Infinity” AFM instrument. The instrument was placed in a clean room with a controlled environment to minimize the environmental noise during measurements. For the measurements, a suitable cantilever with a spring constant of 2 N/m and resonance frequency of 70 kHz was selected to ensure optimal imaging conditions. Before measurements, the AFM instrument was calibrated according to the manufacturer’s recommended calibration procedure. To ensure the reliability of the results, AFM measurements were performed on three regions of each sample, with a scan size area of 40 × 40 μm^2^. For the evaluation of the wavelength and periodicity of the wrinkling pattern, we cross-sectioned each AFM image in six different slices (three horizontal and three vertical) and counted the peaks. Statistical analysis was conducted to assess the variability between the measurements.

### High-Resolution Scanning Electron Microscopy

Before any electron microscopy characterization, all ABS-like samples were coated with a 3 nm iridium layer by using a sputtering coater (Compact Coating Unit 010, Safematic). We used Zeiss Gemini II HR-SEM at an acceleration voltage of 4 keV and working distance of 5 mm using in-lens and Energy-dispersive X-ray (EDS) detectors to characterize the surface morphologies and chemical compositions.

### High-Resolution Transmission Electron Microscopy

Before any electron microscopy characterization, all ABS-like samples were coated with a 3 nm iridium layer using a sputtering coater (Compact Coating Unit 010, Safematic). Cross-sectional transmission electron microscopy (TEM) was used to reveal the morphology and continuity of the oxide layers. We used plasma-focused ion beam milling (Helios 5 PFIB DualBeam, Thermo Fisher) to prepare cross-sectional lamellas. Before the milling, carbon and platinum coatings (∼15 and ∼125 nm, respectively) were deposited to protect the underlying substrates. Images were taken using a high-resolution transmission electron microscope (Titan Themis G2 60-300 FEI, Thermo Fisher) at 200–300 keV, with bright-field TEM and high-angle annular dark field (HAADF) scanning TEM (STEM). EDS STEM with a dual EDS detector was used for elemental mapping.

## Supplementary Material



## Notes

ALD processes data, shear measurements data and mechanical performance, SEM, TEM and AFM characterization, and contact angle measurements (PDF)
