# Supplementary material for: Enhancing the Adhesion Strength of Polymer-Based Joints via Atomic Layer Deposition Surface Modifications
Source: ACS Appl Mater Interfaces. 2025 May 29;17(23):34529–37. doi: 10.1021/acsami.5c04879 (PMC12163925; doi:10.1021/acsami.5c04879)
Supplement: Supplementary file 1 [file am5c04879_si_001.pdf]

Supporting Information

Enhancing the Adhesion Strength of Polymer-Based Joints via Atomic Layer Deposition Surface Modifications

Shachar keren (1), Elina Yachnin (2), Noy Cohen *,(3), Tamar Segal-Peretz *,(1)

Corresponding authors: tamarps@technion.ac.il, noyco@technion.ac.il

(1) Department of Chemical Engineering, Technion–Israel Institute of Technology, Haifa, 3200003, Israel.
(2) Department of Civil and Environmental Engineering, Technion–Israel Institute of Technology, Haifa, 3200003, Israel.
(3) Department of Materials Science and Engineering, Technion–Israel Institute of Technology, Haifa, 3200003, Israel.

ALD processes

Table S1 - ALD processes conditions. All ALD processes were performed at 80°C.

Oxide layer	Precursors	ALD cycles	Average thickness on a silicon wafer (nm)	Growth per cycle (nm)
Al_2O_3	TMA-H_2O	200	14.7±0.1	0.074
		600	46.9±0.1	0.078
		1000	77.7±0.3	0.078
TiO_2	$TiCl_4$-H_2O	200	15.1±0.8	0.076
		600	41.8±1.2	0.070
		1000	83.6±0.4	0.084
ZnO	DEZ-H_2O	200	23.4±0.2	0.117
		600	71.2±0.3	0.119
		1000	121.9±1.2	0.122

Shear Measurements

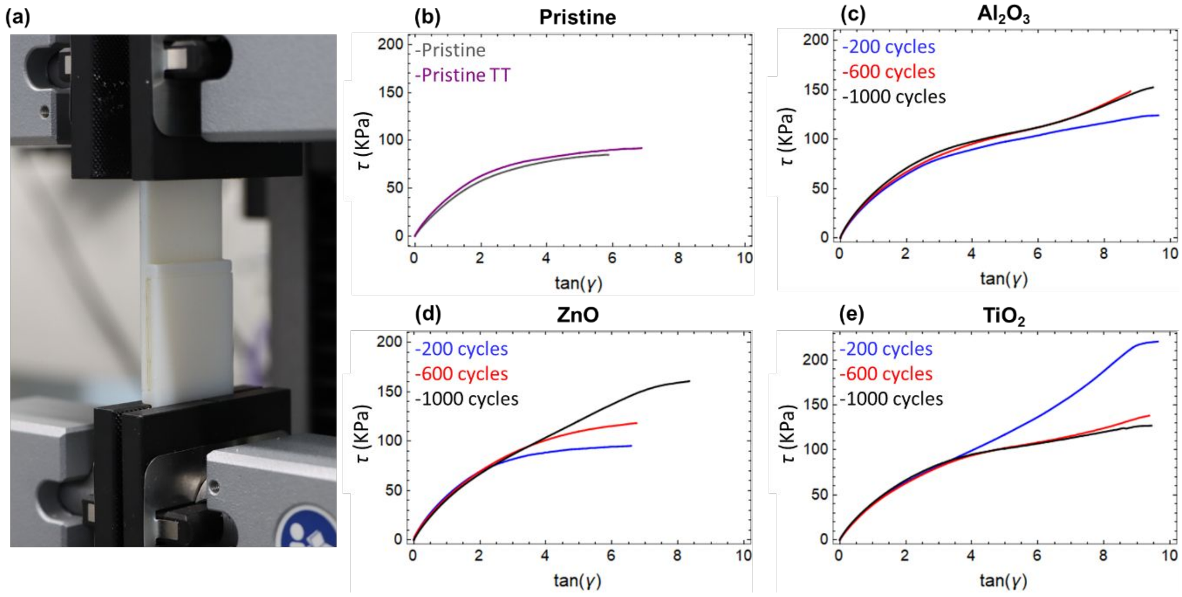

Figure S1 – (a) Single lap shear test setup on tensile machine. (b-d) Shear stress measurements (τ_{max} - shear stress, tan(γ) - strain) for the various ABS samples: (b) pristine ABS with and without thermal treatment (TT), (c) with Al_2O_3 modification layers, (d) with ZnO modification layers and (e) with TiO_2 modification layers. Numeric values of the mechanical properties are shown in Table S2.

Table S2 - Mechanical properties of the pristine and modifies lap shears: shear stress at failure τ_{max}, shear strain at failure $Tan(\gamma)_{max}$, and toughness U_T.

Sample	ALD cycles	τ_{max} (kPa)	$Tan(\gamma)_{max}$	U_T (kJ/m^3)
Pristine	0	85.7±7.1	6.32±0.47	400±56
Pristine TT		92.3±9.7	7.65±0.73	553±115
Al_2O_3	200	124.6±15.1	9.75±0.09	861±75
	600	149.1±11.9	9.40±0.36	880±47
	1000	153.7±32.9	9.74±0.15	965±119
TiO_2	200	220.9±3.8	9.75±0.13	1164±3
	600	139±10.8	9.52±0.12	870±26
	1000	128.1±7.0	9.73±0.23	889±43
ZnO	200	95.3±0.9	6.59±0.00	485±14
	600	108.3±20.3	6.95±0.85	562±135
	1000	163.2±23.2	9.12±0.40	979±123

SEM images

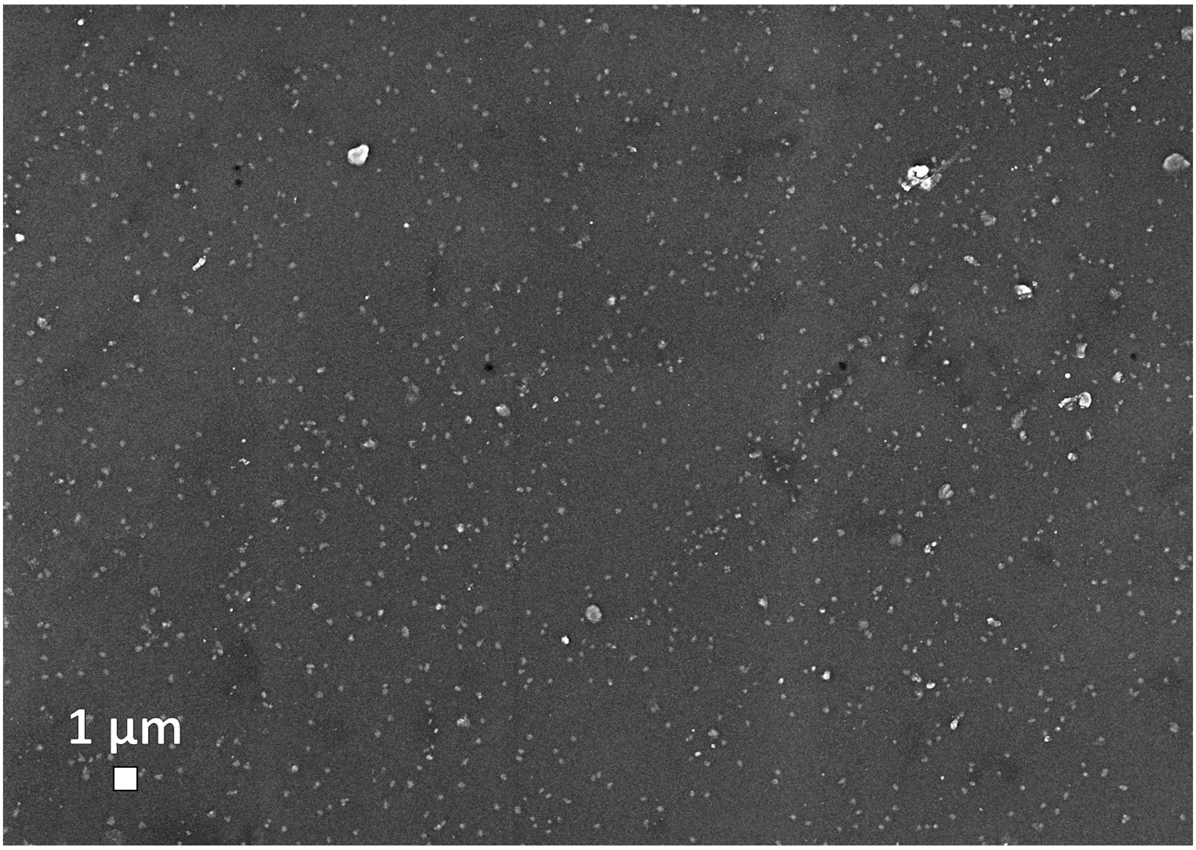

Figure S2 – Top-down SEM images of a pristine ABS substrate (without thermal treatment), demonstrating no wrinkle morphology on the surface.

Contact Angle Measurements

Table S3 - Static contact angle measurements.

Sample	ALD cycles	Contact angle		Surface energy (mJ/m2)		
		Water	Diiodomethane	Polar	Dispersion	Total
Pristine	0	76.2±3.0	11.8±1.0	2.9±0.9	49.7±0.2	52.6±1.1
Pristine TT		77.2±1.5	33.9±1.6	3.9±0.5	42.5±0.7	46.4±1.2
Al_2O_3	200	50.2±2.6	37.9±2.4	17.6±1.5	40.7±1.8	58.2±3.3
	600	46.5±2.5	37.9±1.8	19.7±1.5	40.7±0.9	60.3±2.4
	1000	48.6±1.4	37.7±1.7	18.4±0.9	40.7±0.8	59.2±1.7
TiO_2	200	39.8±1.2	34.0±5.7	22.6±1.4	42.5±2.6	65.1±4.0
	600	62.3±2.5	32.6±1.1	10.1±1.3	43.2±0.5	53.2±1.4
	1000	59.7±4.0	29.1±1.6	11.0±2.0	44.5±0.5	55.5±2.1
ZnO	200	56.4±0.3	31.1±2.7	12.9±0.4	43.8±1.1	56.7±1.5
	600	53.0±2.1	38.8±1.3	16.2±1.2	40.2±0.6	56.4±1.8
	1000	58.4±1.8	33.2±2.0	12.2±1.0	42.8±1.0	55.0±2

AFM Measurements

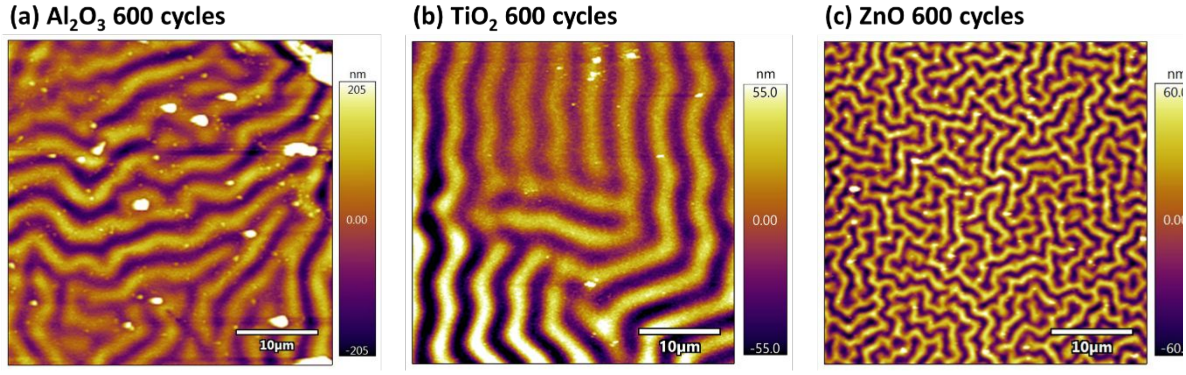

Figure S3 –AFM scans of the various samples after surface modifications of 600 ALD cycles: (a) with Al_2O_3 modification layers, (b) with TiO_2 modification layers and (c) with ZnO modification layers.

Table S4 - The average wavelength of the wrinkle pattern (λ) and amplitudes (A) obtained via AFM scans.

**Substrate**	**ALD cycles**	**λ (μm)**	**A (nm)**
Pristine	0	-	8.7±3.2
Pristine (TT)		-	23.5±12.2
Al_2O_3	200	6.96±1.00	100.5±3.9
	600	10.00±2.04	104.0±34.5
	1000	10.64±2.36	94.5±13.5
TiO_2	200	3.20±0.47	34.4±13.7
	600	6.67±1.81	56.0±28.0
	1000	7.87±1.94	47.0±30.9
ZnO	200	2.11±0.95	6.6±0.3
	600	2.86±0.72	27.7±0.8
	1000	2.26±0.34	64.4±4.1

Table S5 - Poisson's ratios (ν) and elastic moduli (E) of the ABS and various oxides

	**ABS**	**Al_2O_3**	**TiO_2**	**ZnO**
ν	0.36-0.37 [1,2]	0.24 [3]	0.27 [4]	0.36 [5]
***E* (GPa)**	2.6-3 [6]	168-182 [3,7]	151 [4]	143 [8]

TEM Images

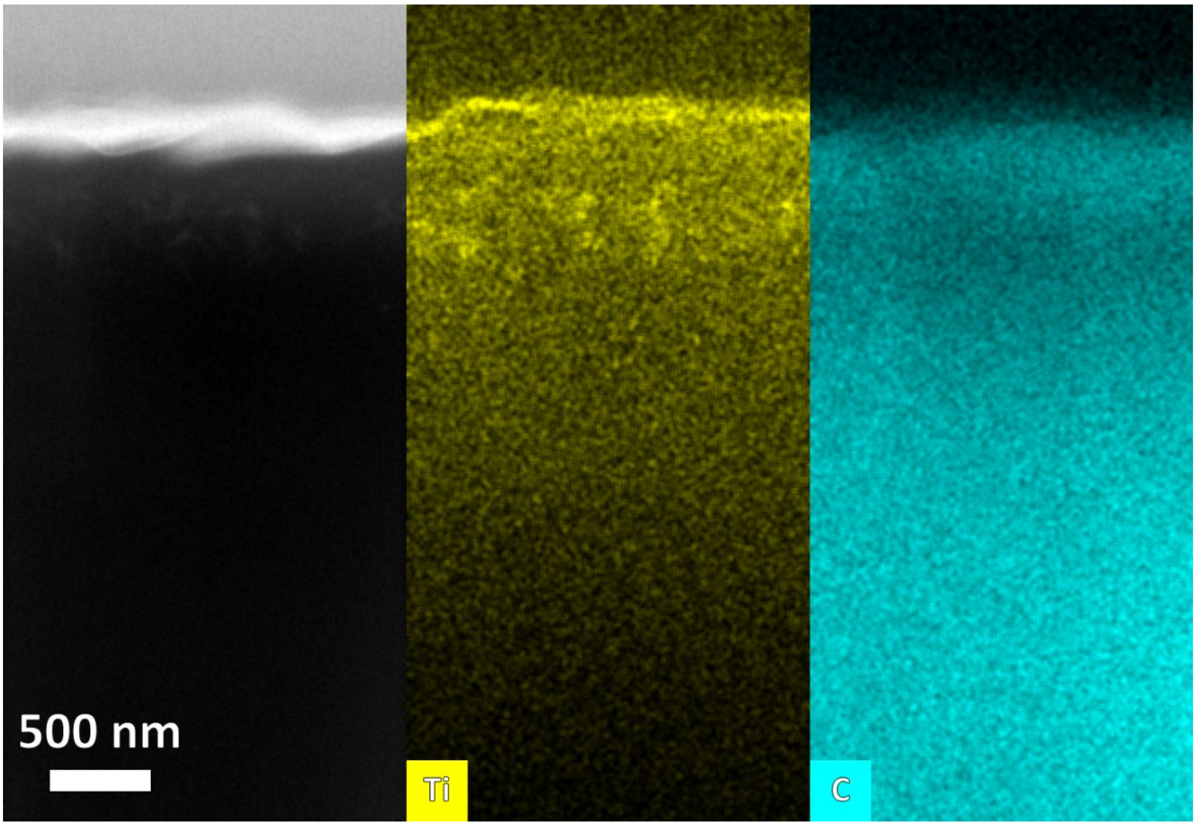

Figure S4 – Cross-sectional TEM-EDS elemental maps of ABS substrates after 800 ALD cycles with ZnO modification layers on top. The comparison between the Zn and the C signals show that ZnO grew mainly inside the ABS layer with infiltration depth of ~2.5 µm and gradient distribution of the ZnO along the infiltration track.

Formation of orchid-like TiO_2 structures

The formation of orchid-shaped structures was observed on the ABS-like surfaces after the growth of TiO_2 with 600 and 1000 cycles (Figure S5a). To examine the nano-structure of the orchid-shaped structures, we cross-sectioned a branch from the structure (see yellow dotted square in Figure S5a), using PFIB milling and then directly probed its spatial morphology using HAADF-STEM combined EDS elemental analysis. The images as shown in Figure S5b reveals a hollow morphology. These structures might have inferior mechanical properties due to the hollow morphology and even low adhesion to the surface, and as a result, they might act as a weak boundary layer and impair adhesion. We note that the formation mechanism of these structures is unclear yet. However, we suspect it might be related to the formation of chloromethane byproducts (see Figure S6).[9]

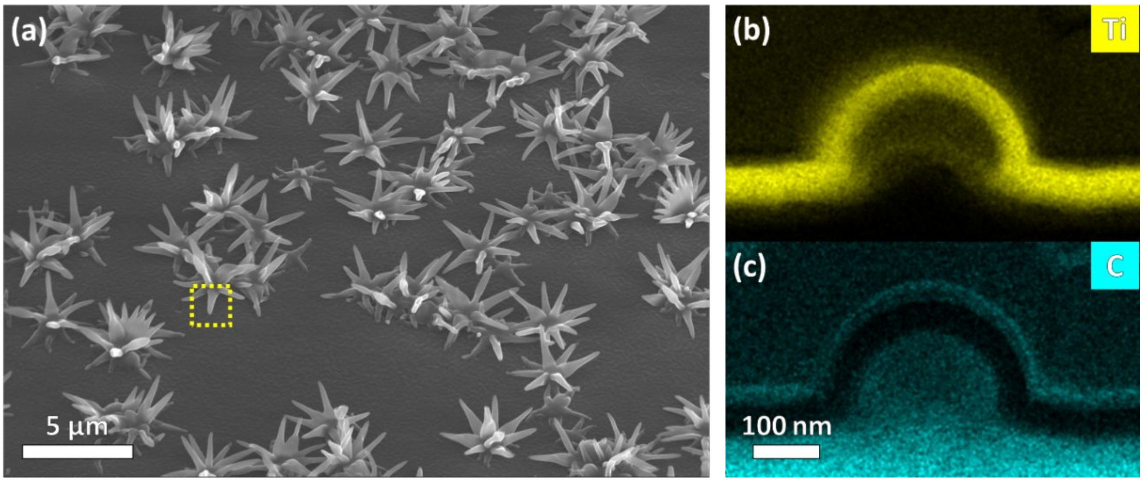

Figure S5 – Electron microscopy of ABS surface after growth of TiO2 with 1000 ALD cycles. (a)A top-down (45° tilt) SEM image. (b-c) Cross-sectional TEM elemental maps of (b) Ti and (c) C of a single branch of an orchid-shaped structure (marked with a dashed square).

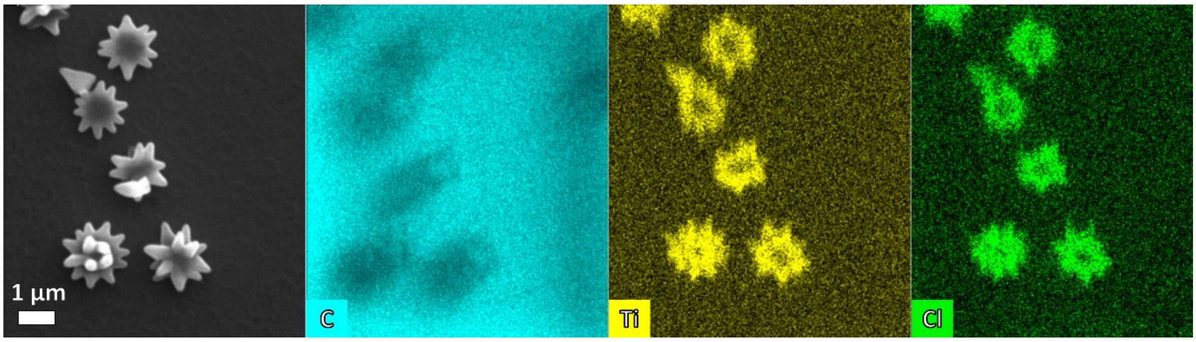

Figure S6 – Top-down SEM images combined with EDS analysis of the orchid-shape structures on top of 1000 ALD cycles TiO2-modified ABS. The orchid-shape structures appear after 600 and 1000 ALD cycles.

1. Zou, R., Xia, Y., Liu, S., Hu, P., Hou, W., Hu, Q., Shan, C., Isotropic and anisotropic elasticity and yielding of 3D printed material. *Composites Part B: Engineering* **99**, 506–513 (2016).

2. Cantrell, J. Experimental Characterization of the Mechanical Properties of 3D-Printed ABS and Polycarbonate Parts, Yoshida, S., Lamberti, L., Sciammarella, C. (eds) *Advancement of Optical Methods in Experimental Mechanics*, **3**. Conference Proceedings of the Society for Experimental Mechanics Series. (2017).

3. Tripp, M. K., Stampfer, C., Miller, D. C., Helbling, T., Herrmann, C. F., Hierold, C., Ken Gall, K., George, S. M., Victor M. Bright, V. M. The mechanical properties of atomic layer deposited alumina for use in micro- and nano-electromechanical systems. *Sensors and Actuators A: Physical* **130–131**, 419–429 (2006).

4. Borgese, L., Gelfi M., Bontempi, E., Goudeau, P., Geandier, G., Thiaudière, D., Depero, L.E. Young modulus and Poisson ratio measurements of TiO2 thin films deposited with Atomic Layer Deposition. *Surface and Coatings Technology* **206**, 2459–2463 (2012).

5. Soomro, M. Y., Hussain, I., Bano, N., Broitman, E., Nur, O., Willander, M. Nanoscale elastic modulus of single horizontal ZnO nanorod using nanoindentation experiment. *Nanoscale Res Lett* **7**, 146 (2012).

6. Digital ABS Plus data sheet. Stratasys.

7. Dusoe, K. J., Ye, X., Kisslinger, K., Stein, A., Lee, S.-W., Nam, C.-Y. Ultrahigh Elastic Strain Energy Storage in Metal-Oxide-Infiltrated Patterned Hybrid Polymer Nanocomposites. *Nano Lett.* **17**, 7416–7423 (2017).

8. Tapily, K., Gu, D., Baumgart, H., Namkoong, G., Stegall, D. and Elmustafa, A. A. Mechanical and structural characterization of atomic layer deposition-based ZnO films. *Semicond. Sci. Technol.* **26**, 115005 (2011).

9. Balogun, S. A., Yim, S. S., Yom, T., Jean, B. C. & Losego, M. D. Dealkylation of Poly(methyl
mophysical Properties of the Hybrid Material. *Chem. Mater*. **36**, 838–847 (2024).
